# Collagenolytic Activities of the Major Secreted Cathepsin L Peptidases Involved in the Virulence of the Helminth Pathogen, *Fasciola hepatica*

**DOI:** 10.1371/journal.pntd.0001012

**Published:** 2011-04-05

**Authors:** Mark W. Robinson, Ileana Corvo, Peter M. Jones, Anthony M. George, Matthew P. Padula, Joyce To, Martin Cancela, Gabriel Rinaldi, Jose F. Tort, Leda Roche, John P. Dalton

**Affiliations:** 1 Infection, Immunity and Innovation (i3) Institute, University of Technology Sydney (UTS), Sydney, New South Wales, Australia; 2 Departmento de Genética, Facultad de Medicina, Universidad de la República (UDELAR), Montevideo, Uruguay; 3 Institute of Parasitology, McGill University, St. Anne de Bellevue, Quebec, Canada; University of Queensland, Australia

## Abstract

**Background:**

The temporal expression and secretion of distinct members of a family of virulence-associated cathepsin L cysteine peptidases (FhCL) correlates with the entry and migration of the helminth pathogen *Fasciola hepatica* in the host. Thus, infective larvae traversing the gut wall secrete cathepsin L3 (FhCL3), liver migrating juvenile parasites secrete both FhCL1 and FhCL2 while the mature bile duct parasites, which are obligate blood feeders, secrete predominantly FhCL1 but also FhCL2.

**Methodology/Principal Findings:**

Here we show that FhCL1, FhCL2 and FhCL3 exhibit differences in their kinetic parameters towards a range of peptide substrates. Uniquely, FhCL2 and FhCL3 readily cleave substrates with Pro in the P2 position and peptide substrates mimicking the repeating Gly-Pro-Xaa motifs that occur within the primary sequence of collagen. FhCL1, FhCL2 and FhCL3 hydrolysed native type I and II collagen at neutral pH but while FhCL1 cleaved only non-collagenous (NC, non-Gly-X-Y) domains FhCL2 and FhCL3 exhibited collagenase activity by cleaving at multiple sites within the α1 and α2 triple helix regions (Col domains). Molecular simulations created for FhCL1, FhCL2 and FhCL3 complexed to various seven-residue peptides supports the idea that Trp67 and Tyr67 in the S2 subsite of the active sites of FhCL3 and FhCL2, respectively, are critical to conferring the unique collagenase-like activity to these enzymes by accommodating either Gly or Pro residues at P2 in the substrate. The data also suggests that FhCL3 accommodates hydroxyproline (Hyp)-Gly at P3-P2 better than FhCL2 explaining the observed greater ability of FhCL3 to digest type I and II collagens compared to FhCL2 and why these enzymes cleave at different positions within the Col domains.

**Conclusions/Significance:**

These studies further our understanding of how this helminth parasite regulates peptidase expression to ensure infection, migration and establishment in host tissues.

## Author Summary

*Fasciola hepatica* is a helminth parasite that causes liver fluke disease (fasciolosis) in domestic animals (sheep and cattle) and humans worldwide. In order to infect their mammalian hosts, *F. hepatica* larvae must penetrate and traverse the intestinal wall of the duodenum, move through the peritoneum and penetrate the liver. After migrating through the liver, causing extensive tissue damage, the parasites move to their final niche in the bile ducts where they mature and feed on host haemoglobin to support the production of eggs. To achieve these tasks, *F. hepatica* secretes a number of distinct cathepsin L cysteine peptidases (FhCL). Thus, the infective larvae that penetrate the host gut secrete cathepsin L3 (FhCL3), the migrating liver-stage juvenile parasites secrete both FhCL1 and FhCL2 while mature bile duct parasites that feed on host blood secrete predominantly FhCL1 but also FhCL2. Here we show that the major cathepsin L peptidases secreted by *F. hepatica* (FhCL1, FhCL2 and FhCL3) display differential ability to degrade host collagen (an important component of host tissues) and investigate this phenomenon at the molecular level.

## Introduction

Papain-like cysteine peptidases, including cathepsins B and L, are ubiquitously secreted extracorporeally by helminth parasites of human and veterinary importance where they perform many important roles that are critical to the development and survival of the parasite within the mammalian host [1]. These roles include penetration and migration through host tissues [2], catabolism of host proteins to peptides and amino acids [3], [4], and modulation of the host immune response by cleaving immunoglobulin [5], [6] or by altering the activity of immune effector cells [7]. Accordingly, cathepsin peptidases are leading targets for novel anti-parasitic drugs and vaccines that block their function [8], [9].

*Fasciola hepatica* is the causative agent of liver fluke disease (fasciolosis) of domestic animals in regions with temperate climates. Although traditionally regarded as a disease of livestock, fasciolosis is now recognised as an important emerging foodborne zoonotic disease in rural areas of South America (particularly Bolivia, Peru and Equador), Egypt and Iran [10]. It is estimated that over 2.4 million people are infected with *F. hepatica* worldwide and around 91 million are at risk of infection [11]. To infect their mammalian hosts, *F. hepatica* larvae, which are ingested with vegetation contaminated with dormant cysts (metacercariae), penetrate the intestinal wall, enter the liver capsule and migrate through the parenchyma before invading into the bile ducts [12]. To facilitate this tissue migration, *Fasciola* secrete various members of a multigenic family of cathepsin L peptidases that exhibit overlapping but complementary substrate specificities and together cleave host macromolecules very efficiently [13], [14]. In fact, the ability of *Fasciola* to infect and adapt to a wide range of host species has been attributed to the effectiveness of this proteolytic machinery [14], [15].

Phylogenetic analyses have shown that the *Fasciola* cathepsin L gene family expanded by a series of gene duplications followed by divergence which gave rise to three clades expressed by tissue-migrating and adult worms (Clades 1, 2, and 5) and two clades specific to the early infective juvenile stage (Clades 3 and 4) [13], [14]. Consistent with these observations, our proteomics analysis identified representative enzymes from Clades 1, 2 and 5, but not from Clades 3 and 4, in the secretory products of adult *F. hepatica* [14]. More recently, we showed that the temporal expression and secretion of the specific cathepsin L clades correlated with the migration of the parasite through host issues; members of cathepsin L clade 3 (FhCL3) are secreted by *Fasciola* infective larvae and effected penetration of the host intestinal wall while clades 1, 2 and 5 (FhCL1, FhCL2 and FhCL5) peptidases are secreted by the immature liver-stage flukes and adult worms and function in preparing a migratory path through the liver and in the acquisition of nutrient by degrading host blood and tissue components. While clade 4 (FhCL4) peptidases are expressed by infective larvae they do not seem to be secreted and, therefore, may play an intracellular house-keeping function [13], [16]. Recent transcriptomic analyses of juvenile and adult stages have confirmed these observations [17], [18].

The secreted *Fasciola* cathepsins are produced in specialised gastrodermal cells which line the parasites's gut and are packaged in secretory vesicles before being extruded into the gut lumen from where they are released into host tissues [19]. The peptidases can efficiently degrade a range of host macromolecules including haemoglobin, immunoglobulin and interstitial matrix proteins such as fibronectin and laminin [3], [4], [20]–[22]. Notably, however, studies in our laboratory using functionally-active recombinant enzymes have shown that FhCL2 and FhCL3 exhibit an unusual ability to cleave native collagen [22], [23]. This is of relevance because collagenase-like activity is restricted to very few enzymes (e.g. bacterial collagenases, matrix metalloproteinases and human cathepsin K) and, therefore, the evolution and maintenance of such an activity in *Fasciola* suggests that it is essential to the parasite's ability to degrade the connective tissue matrix of the organs through which it migrates.

The active site of papain-like cysteine peptidases is relatively short, and while consisting of four subsites (S2-S1-S1′-S2′) with additional binding areas (S4-S3 and S3′) the specificity of substrate binding is principally governed by the residues that make up the S2 subsite [24], [25]. This S2 site forms a deep pocket capable of holding the P2 amino acid of the substrate and positioning the scissile bond into the S1 subsite for cleavage. In *Carica papaya* papain (PDB ID: 9PAP), the S2 subsite is composed of residues occupying positions 67, 68, 133, 157, 160 and 205. An analysis of these residues in the various cathepsin L clades clearly demonstrates divergence within the S2 subsite, in particular at the three positions that have the greatest influence on P2 binding i.e. at residues 67, 157 and 205 [14], [15], [23]. For FhCL2, the collagenolytic activity has been attributed to the presence of a particular residue, Tyr69, within the enzyme's S2 substrate binding site which is also found in human cathepsin K, the only mammalian cathepsin with the ability to cleave within the covalently-linked triple helices, of Col domains of native collagen [26], [27]. The S2 Tyr69 is also suggested to allow both enzymes to cleave macromolecular and dipeptide substrates with a Pro residue in the P2 position [22], [28]. More recently we showed that in FhCL3, this position is occupied by a larger Trp residue, a feature shared only with a ginger rhizome peptidase [29] which is also capable of cleaving collagen. Notably the S3 subsite of both FhCL3 and the plant enzyme are quite shallow, an observation that led us to advance the idea that the specificity of these enzymes might be restricted [23].

Our laboratory recently determined the three-dimensional structure of one of the major cathepsin L peptidases of adult *F. hepatica*, FhCL1 [22]. Similar to other cathepsins, the enzyme is composed of two domains (R and L) at the juncture of which is a cleft that forms the substrate-binding site and contains the enzyme catalytic machinery. Super-imposition of the alpha carbons of FhCL1 with cysteine peptidases from plants (e.g. papain, PDB ID 9PAP) and mammals (e.g. human cathepsin L PDB ID 1CJL) yields an r.m.s. deviation in the range 0.78 Å to 1.085 Å which is indicative of the very high conservation of the overall fold and shape that exists amongst all members of the papain family of cysteine peptidases [30]. Since the FhCL1 structure and fold can be described as practically identical to all other cathepsin L-like peptidases its scaffold can be exploited as a ‘prototype’ to investigate the role of critical amino acids within the S_2_ subsite in substrate binding (Table 1), particularly those of FhCL2 and FhCL3, whose primary structures are 78% and 70% identical to FhCL1, respectively. In the present study, we investigated and compared the substrate specificity of active recombinant forms of FhCL1, FhCL2 and FhCL3 with specific emphasis on their ability to degrade native collagen. This activity was interpreted by obtaining the enzymatic kinetic parameters (*Km*, *k*cat, and *k*cat/*Km*) of these enzymes on a range of peptide substrates and binding kinetics for specific inhibitory compounds. Furthermore, using mass spectrometry we mapped the cleavage sites of native collagen I and derived peptides, adding evidence for differential activities between FhCL2 and FhCL3. Finally, using our FhCL1 crystal structure as a template we created molecular dynamics simulations to explain how the active sites of these enzymes accommodate collagen-like substrates and endow them with this unusual collagenolytic activity. Our study provides biochemical and structural insights into the molecular mechanism of tissue invasion by these important parasitic helminths.

**Table 1:** Residues forming the S2 active site of human and *F. hepatica* cathepsin L peptidases.

	Residues					
	67	68	133	157	160	205
Human cathepsin L	Leu	Met	Ala	Met	Gly	Ala
*C. papaya* papain	Tyr	Pro	Val	Val	Ala	Ser
FhCL1	Leu	Met	Ala	Val	Ala	Leu
FhCL2	Tyr	Met	Ala	Leu	Ala	Leu
FhCL3	Trp	Met	Ala	Val	Ala	Val

Comparison of the residues from the S2 active site that contribute to differential substrate-binding in human cathepsin L, *C. papaya* papain and *F. hepatica* cathepsins FhCL1, FhCL2 and FhCL3.

## Materials and Methods

### Materials

Z-Phe-Arg-NHMec, Z-Leu-Arg-NHMec, Z-Val-Val-Arg-NHMec, Tos-Gly-Pro-Arg-NMec, Tos-Gly-Pro-Lys-NMec, Boc-Ala-Gly-Pro-Arg-NMec, Boc-Val-Leu-Lys-NMec, Boc-Val-Pro-Arg-NMec, Z-Phe-Ala-CHN2, Z-Gly-Pro-Gly-Gly-Pro-Ala and Z-Gly-Pro-Leu-Gly-Pro were obtained from Bachem (St. Helens, UK). Cathepsin K inhibitor II was purchased from BD Biosciences (Sydney, Australia). E-64, DTT, EDTA and bovine nasal septum collagen type II were obtained from Sigma-Aldrich (Sydney, Australia). Calf skin collagen type I was purchased from Calbiochem. *Pichia pastoris* strain X33 was obtained from Invitrogen (San Diego, CA, USA). Ni-NTA agarose and columns were obtained from Qiagen (Australia). Pre-cast NuPage 4–12% Bis-Tris gels and pre-stained molecular weight markers were purchased from Invitrogen (Australia).

### Expression and purification of recombinant *F. hepatica* cathepsins in yeast

Recombinant *F. hepatica* procathepsin L1, L2 and L3 (FhCL1, FhCL2 and FhCL3) were produced in yeast as previously described [22], [23]. Briefly, *P. pastoris* (for FhCL1 and FhCL2 expression) and *P. angusta* (for FhCL3 expression) yeast transformants were cultured in 500 ml BMGY broth, buffered to pH 8.0, in 5 L baffled flasks at 30°C until an OD_600_ of 2–6 was reached. Cells were harvested by centrifugation at 2000× g for 5 min and protein expression induced by resuspending in 100 ml BMMY broth, buffered at pH 6.0 containing 1% methanol. Recombinant proteins were affinity purified from yeast using Ni-NTA-agarose. Recombinant propeptidases were dialysed against phosphate buffered saline (PBS) and stored at −20°C. The 37 kDa cathepsin L zymogens were autocatalytically activated and processed to 24.5 kDa mature enzymes by incubation for 2 h at 37°C in 0.1 M sodium citrate buffer (pH 5.0) containing 2 mM DTT and 2.5 mM EDTA. The mixture was then dialysed against PBS, pH 7.3. The proportion of functionally active recombinant protein in these preparations was determined by titration against E-64.

### Enzyme assays and kinetics with fluorogenic peptide substrates

Initial rates of hydrolysis of the fluorogenic peptide substrates shown in table 2 were monitored by the release of the fluorogenic leaving group, NHMec, at an excitation wavelength of 380 nm and an emission wavelength of 460 nm using a Bio-Tek KC4 microfluorometer. *k*cat and *Km* values were determined using nonlinear regression analysis. Initial rates were obtained at 37°C over a range of substrate concentrations spanning *Km* values (0.2–200 µM) and at fixed enzyme concentrations (0.5–5 nM). Assays were performed in 100 mM sodium phosphate buffer (pH 6.0) containing 1 mM DTT and 1 mM EDTA. Rate constants for the inactivation of the *Fasciola* enzymes by Z-Phe-Ala-CHN2 and cathepsin K inhibitor II were determined from progress curves in the presence of substrate as previously described [22].

**Table 2:** Kinetic parameters for hydrolysis of peptidyl-NHMec substrates by recombinant *F. hepatica* cathepsin Ls.

Enzyme	Substrate	K_M_ µM	±	Kcat s^−1^	±	Kcat/K_M_ Ms^−1^
FhCL1	Z-LR-NHMec	1.09	0.38	1.63	0.1	1492354.7
FhCL2	Z-LR-NHMec	2.13	0.29	0.95	0.035	444106.1
FhCL3	Z-LR-NHMec	48	5.7	1.2	0.05	25000
FhCL1	Z-FR-NHMec	1.9	0.57	0.12	0.008	64912.3
FhCL2	Z-FR-NHMec	7.8	1.5	0.09	0.008	11088
FhCL3	Z-FR-NHMec	20.05	2.9	0.02	0.001	999.3
FhCL1	Boc-VLK-NHMec	2.5	0.6	0.14	0.007	54266.7
FhCL2	Boc-VLK-NHMec	2.2	0.79	0.07	0.005	30712.5
FhCL3	Boc-VLK-NHMec	6.13	1.6	0.01	0.001	1747.8
FhCL1	Z-VVR-NHMec	2.84	0.7	0.021	0.001	7464.8
FhCL2	Z-VVR-NHMec	1.53	0.29	0.014	0.001	9237.5
FhCL3	Z-VVR-NHMec	11.7	2.1	0.043	0.020	3696.6
FhCL1	Tos-GPR-NHMec	27.2	4.9	0.02	0.001	671.6
FhCL2	Tos-GPR-NHMec	13.9	3.2	0.26	0.02	18559.5
FhCL3	Tos-GPR-NHMec	10.6	2.3	1.015	0.08	95774.7
FhCL1	Tos-GPK-NHMec	17.42	2.6	0.01	0.001	612.3
FhCL2	Tos-GPK-NHMec	12.9	3.4	0.18	0.013	13746.8
FhCL3	Tos-GPK-NHMec	9.8	3.7	0.15	0.015	36419.8
FhCL1	Boc-VPR-NHMec	22	2.5	0.01	0.0004	478.8
FhCL2	Boc-VPR-NHMec	11.39	2.1	0.23	0.01	20193.2
FhCL3	Boc-VPR-NHMec	21.1	3.2	0.033	0.002	1564
FhCL1	Boc-AGPR-NHMec	18.8	4	0.01	0.001	673.8
FhCL2	Boc-AGPR-NHMec	9.4	1.3	0.55	0.023	58027.1
FhCL3	Boc-AGPR-NHMec	9.6	1.6	0.58	0.025	60763.9

### Hydrolysis of native collagen and collagen-like peptides

Calf skin collagen type I and bovine nasal septum collagen type II (solubilised in 0.2 M acetic acid at a concentration of 2 mg/ml) were dialysed for two days against 0.1 M sodium acetate (pH 5.5) or PBS (pH 7.0). Digestion reactions contained 10 µg of dialysed collagen substrates, 1 mM DTT and 1 mM EDTA and 2 µM activated FhCL1, FhCL2 or FhCL3 in a final volume of 100 µl of one of the above buffers at 28°C. For collagen type 1, reactions were performed for 3 h (pH 5.5) or 20 h (pH 7.0) whilst collagen type II was digested over 13–18 h. All reactions were stopped by the addition of 10 µM E-64. Digests were analyzed on reducing 4–12% NuPage Bis-Tris gels and visualised by staining with Flamingo fluorescent stain (Bio-Rad).

For digestion of collagen-like peptide substrates, 20 µg of Z-Gly-Pro-Leu-Gly-Pro and Z-Gly-Pro-Gly-Gly-Pro-Ala in DMSO were incubated with FhCL2 or FhCL3 (15 µM) in 100 mM sodium acetate buffer, pH 4.5, containing 1 mM EDTA and 2 µM DTT for 30 min at 37°C. Digestion reactions were halted by the addition of 10 µM E-64.

### Analysis of collagen digests by mass spectrometry

Recombinant FhCL2 and FhCL3 were removed from collagen type I digests using Ni-NTA agarose. The reactions were then spun at 13,000 rpm for 15 min to remove particulates and were concentrated to a final volume of 15 µl using a Concentrator 5301 (Eppendorf). Using an Eksigent AS-1 autosampler connected to a Tempo nanoLC system (Eksigent, USA), 10 µL of the sample was loaded at 20 µl/min with MS buffer A (2% acetonitrile+0.2% formic acid) onto a C8 trap column (Michrom, USA). After washing the trap for three minutes, the peptides were washed off the trap at 300 nL/min onto an IntegraFrit column (75 µm×100 mm) packed with ProteoPep II C18 resin (New Objective, Woburn, MA). Peptides were eluted from the column and into the source of a QSTAR Elite hybrid quadrupole-time-of-flight mass spectrometer (AB Sciex) using the following program: 5–50% MS buffer B (98% acetonitrile+0.2% formic acid) over 15 minutes, 50–80% MS buffer B over 5 minutes, 80% MS buffer B for 2 minutes, 80–5% for 3 min. The eluting peptides were ionised with a 75 µm ID emitter tip that tapered to 15 µm (New Objective) at 2300 V. An Intelligent Data Acquisition (IDA) experiment was performed, with a mass range of 375–1500 Da continuously scanned for peptides of charge state 2+–5+ with an intensity of more than 30 counts/s. Selected peptides were fragmented and the product ion fragment masses measured over a mass range of 50–1500 Da. The mass of the precursor peptide was then excluded for 15 seconds. Peak list files generated by MSX (Infochromics) were exported to a local PEAKS Studio v5.0 (Bioinformatics Solutions Inc.) search engine for protein database searching. MS/MS data was used to search a custom-made database containing only bovine collagen sequences. The enzyme specificity was set to “no enzyme” and propionamide (acrylamide) modification of cysteines was used as a fixed parameter and oxidation of methionines was set as a variable protein modification. The mass tolerance was set at 100 ppm for precursor ions and 0.2 Da for fragment ions. Only 1 missed cleavage was allowed. Matched peptides achieving a score >60% were accepted during PEAKs searches [16]. The matching peptides were then mapped onto the primary amino acid sequence of bovine collagen to identify FhCL2 and FhCL3 cleavage sites and to plot P2 residue preference for each enzyme.

For collagen-like peptide substrates, digests were concentrated and analysed by MS/MS as described above with the following modifications. The mass range of 150–600 Da was scanned for peptides of charge state 2+ with an intensity of more than 100 counts/s. Selected peptides were fragmented and the product ion fragment masses measured over a mass range of 50–600 Da. The mass of the precursor peptide was then excluded for 120 seconds. An inclusion list describing all possible substrate ions that could be produced by enzymatic cleavage of the peptide substrates was generated and programmed into the Analyst acquisition software. The resulting data files were manually interrogated to determine the presence of peptide ions described in the inclusion list. The MS/MS spectra of those peptides were *de novo* sequenced for b and y ion fragments describing the peptide substrate's sequence to a mass accuracy of approximately 0.2 Da.

### Molecular dynamics (MD) simulations

For the MD simulations, starting coordinates for *F. hepatica* cathepsin L were taken from the 1.4 Å resolution crystal structure of a FhCL1 mutant zymogen, in which the active site Cys was replaced by Gly ([22]; PDB 2O6X). The prosegment (residues 1–100) was removed and the active site Gly mutation reversed to the wild type Cys. Initial coordinates for a template peptide substrate (Ala-Leu-Ala-Leu-Pro) were derived from X-ray structures of inhibitors bound to human cathepsin K ([31]; PDB 1NLJ) and bovine cathepsin B ([32]; PDB 1SP4) after structural alignment with FhCL1. This initial peptide was altered to Ala-Leu-Arg-Asn-Ala using the mutate function in Swiss-PdbViewer ([33]; http://spdbv.vital-it.ch/) and then minimized while bound to the wild-type FhCL1 using the equilibration protocol described below. The equilibrated peptide was then extended by one Ala residue at its N- and C-termini using the coordinate generation function in the psfgen program [34], and then re-equilibrated. The resultant peptide (ligand A) was used to generate all other substrate starting coordinates by using the mutate function in Swiss-PdbViewer. Mutations to FhCL1 were similarly generated using Swiss-PdbViewer. Rotamers for mutated enzyme and substrate side-chains were chosen by visual inspection and using the rotamer score provided in Swiss-PdbViewer. The N-terminal residue of the substrate was acetylated and the C-terminus N-methylamidated. Each complex was optimally oriented to minimize cell volume [35] and solvated in a truncated octahedral periodic cell with a minimum of 20 Å between periodic images of the protein. The system was neutralized with sodium ions.

MD simulations were carried out with NAMD 2.6 [34] using the CHARMM27 force field with ϕ/ψ cross-term map corrections [36]. Parameters for Hyp were from Veld and Stevens [37]. Water molecules were simulated with the TIP3P model [38]. Simulation conditions were maintained at 1.0 atm constant pressure by the Nosé-Hoover Langevin piston method [39], [40] and at 310 K constant temperature by Langevin dynamics with a damping coefficient at 5 ps^−1^. The time step used for the simulations was 1.5 fs. A cutoff of 12 Å, with a switching function between 10 and 12 Å, was used for short-range non-bonded interactions. Long-range electrostatic interactions were computed using the particle mesh Ewald method [41] with a grid density of approximately 1/Å. A multiple time-stepping algorithm was used with interactions involving covalent bonds and short-range non-bonded interactions computed every time step, while long-range electrostatic forces were computed every two time steps. SHAKE [42] and SETTLE [43] were applied to constrain the lengths of all bonds that involve hydrogen.

The solvated starting structure was minimized using conjugate gradient minimization to a 0.5 kcal/(mol·Å) r.m.s. gradient with all enzyme heavy atoms fixed, with the exception of side-chain atoms of mutated residues, which were unrestrained. In addition, in this phase of the equilibration, ligand atoms were not fixed and harmonic positional constraints of 100 kcal/(mol·Å^2^) force constant were placed on the Cα atoms of ligand residues 3–5 (P2, P1 and P1′). The unrestrained atoms were then further minimized during a 50 ps molecular dynamics run at 310 K. This starting model was then minimized with harmonic positional constraints on the NCαCO backbone of the protein and Cα atoms of ligand residues 3–5. A 100 kcal/(mol·Å^2^) force constant was used to minimise the system to a 0.5 kcal/(mol·Å) r.m.s. gradient. The constraints were gradually removed by subsequent minimizations to a 0.1 kcal/(mol·Å) r.m.s. gradient, scaling the initial force constants by factors of 0.5, 0.15, 0.05, and 0. The unrestrained minimized structure was then heated from 50 K to 310 K in steps of 25 K using velocity reassignment during a 30 ps molecular dynamics run. The equilibrated system was then used for production runs with no restraints. All systems were run for 12 ns. All simulations remained stable to completion. For analysis, the distance between the sulphur atom of the active Cys residue and the scissile backbone carbonyl carbon of the substrate (S-C distance) was recorded every 50 time-steps (0.075 ps); trajectory coordinates were recorded every 1000 time-steps (1.5 ps).

### Free energy of binding calculations

The free energy of binding of the peptide ligand to the peptidase contains an enthalpic and an entropic contribution. Free energy analysis of the production trajectories employed the single-trajectory MM/PBSA method combined with a determination of the change in configurational entropy using the harmonic approximation of normal-mode analysis [44], [45]. Snapshots from the MD trajectory, stripped of water and counterions, were analysed. The enthalpy of binding is composed of the change in the molecular mechanics free energy upon complex formation, and the solvated free energy contribution. The molecular mechanics free energy difference was calculated using the SANDER module in AMBER 9 [46], with no cutoff for the non-bonded energies and the AMBER ff03 force field to describe the protein and peptide ligands [47]. Compatible parameters for Hyp were not available and binding energies for ligand F were not calculated. The AMBER PBSA module was used for the evaluation of the electrostatic free energy of solvation. A grid density of 3/Å was employed for the cubic lattice, the internal and external dielectric constants were set to 1 and 80, respectively, and 1000 linear iterations were performed. The non-polar solvation free energy was calculated from the solvent accessible surface area using the MSMS program [48], with a probe radius of 1.4 Å, the surface tension set to 0.00542 kcal/(mol·Å^2^), and the off-set to 0.92 kcal/mol·m.

The changes in configurational entropy upon ligand association were estimated by an all-atom normal-mode analysis performed with the AMBER NMODE module. Prior to the normal mode calculations, the complex, receptor, and ligand were subjected to minimization with a distance dependent dielectric constant 4*r* and convergence tolerance tighter than a root-mean-squared gradient of drms 10^−4^ kcal/(mol·Å). Entropy and enthalpy calculations on all peptidase-ligand systems are performed separately and averaged over equally spaced snapshots, extracted over the final 4.005 ns of the production phase. The mean of the binding free enthalpies and entropies of all the snapshots were computed and then summed to obtain the binding free energy. For the enthalpy calculations, snapshots were taken every 10.5 ps (381 frames), for the entropy calculations, snapshots were taken every 190.5 ps (21 frames). VMD [49] was used to prepare the initial simulation system and analyse trajectories. Structural figures were prepared with PyMol [50]. Simulaid (http://atlas.physbio.mssm.edu/~mezei/) was used in the preparation of the truncated octahedral cell [35] and to convert the NAMD dcd format MD coordinate trajectories to AMBER format for the MM/PBSA analysis.

## Results and Discussion

### *F. hepatica* FhCL1, FhCL2 and FhCL3 exhibit distinct substrate specificities

Functionally active recombinant forms of the major cathepsin L peptidases of *F. hepatica*, FhCL1, FhCL2 and FhCL3, were expressed in yeast and isolated to homogeneity as previously described [22], [23]. To compare their biochemical substrate specificity the kinetic parameters (*Km*, *k*cat, and *k*cat/*Km*) for each enzyme was determined against a range of small fluorogenic peptide (predominantly tripeptide) substrates (Table 2).

FhCL1 most efficiently cleaved substrates containing hydrophobic residues at the P2 position such as the dipeptides Z-Leu-Arg-NHMec (*k*cat/*Km* 1,492,354 M^−1^ s^−1^), Z-Phe-Arg-NHMec (*k*cat/*Km* 64,912 M^−1^ s^−1^) and tripeptide Boc-Val-Leu-Lys-NHMec (*k*cat/*Km* 54,266 M^−1^ s^−1^). In contrast, tripeptide substrates containing Pro at the P2 position, including Tos-Gly-Pro-Arg-NHMec (*k*cat/*Km* 671 M^−1^ s^−1^), Boc-Ala-Gly-Pro-Arg-NHMec (*k*cat/*Km* 673 M^−1^ s^−1^), Tos-Gly-Pro-Lys-NHMec (*k*cat/*Km* 612 M^−1^ s^−1^) and Boc-Val-Pro-Arg (*k*cat/*Km* 478 M^−1^ s^−1^), were cleaved relatively poorly (Table 2).

In comparison to FhCL1, substrates with Phe and Leu in the P2 position were much less effectively cleaved by FhCL2 and even less so by FhCL3. The *k*cat/*Km* values for FhCL2 and FhCL3 against Z-Phe-Arg-NHMec were 6- and 65-fold lower, respectively, than that observed for FhCL1. Similarly, the *k*cat/*Km* values for Z-Leu-Arg-NHMec were 3.5- and 66-fold lower than FhCL1 for FhCL2 and FhCL3 respectively. By contrast, FhCL2 and FhCL3 cleaved Pro-containing substrates much more readily than FhCL1 with *k*cat/*Km* values of 18,559 M^−1^ s^−1^ (28-fold greater, FhCL2) and 95,774 M^−1^ s^−1^ (142-fold increase, FhCL3) for Tos-Gly-Pro-Arg-NHMec; 58,027 M^−1^ s^−1^ (86-fold increase, FhCL2) and 60,763 M^−1^ s^−1^ (90-fold increase, FhCL3) for Boc-Ala-Gly-Pro-Arg-NHMec; 13,746 M^−1^ s^−1^ (22-fold increase, FhCL2) and 36,419 M^−1^ s^−1^ (60-fold increase, FhCL3) for Tos-Gly-Pro-Lys-NHMec and 21,193 M^−1^ s^−1^ (44-fold increase, FhCL2) and 1,564 M^−1^ s^−1^ (3-fold increase, FhCL3) for Boc-Val-Pro-Arg-NHMec (Table 2). Collectively, these data highlight significant differences in the substrate specificity of the three major *F. hepatica* cathepsin L peptidases. More specifically, the data demonstrates that FhCL3 prefers a bulky Pro residue in the P2 position of substrates over hydrophobic residues such as Leu or Phe, while FhCL2 can readily accept Pro despite preferring hydrophobic moieties at P2, and FhCL1 has an inverse preference to FhCL3.

### Kinetic analyses of recombinant *F. hepatica* peptidases with specific inhibitors

Peptidyl diazomethyl ketones are irreversible inhibitors of cysteine peptidases [51]. Changes in rates of inactivation by these inhibitors have highlighted different specificities at subsites of cysteine peptidases such as cathepsin L and cathepsin B [52]. In this study, we measured the rates of inactivation of FhCL1, FhCL2 and FhCL3 by the cathepsin inhibitor Z-Phe-Ala-CHN_2_. Both FhCL1 and FhCL2 were rapidly inactivated by Z-Phe-Ala-CHN_2_ with the rate of inactivation of FhCL1 being almost 2-fold higher than that of FhCL2 (Table 3). This is in accordance with our previous data [22] and demonstrates that FhCL1 accommodates hydrophobic P2 residues more effectively than FhCL2. In contrast, the rate of inactivation of FhCL3 by Z-Phe-Ala-CHN_2_ was 20-fold times lower, showing that Z-Phe-Ala-CHN_2_ is a poor inhibitor of FhCL3 (Table 3). This is in agreement with our kinetic substrate data using peptidyl fluorogenic substrates (Table 2) that revealed the poor capacity of FhCL3 to accommodate hydrophobic residues in the P2 position.

**Table 3:** Inhibition parameters of Z-Phe-Ala-CHN_2_ and cathepsin K inhibitor II against recombinant *F. hepatica* cathepsin Ls.

Enzyme	Inhibitor	Ki_(app)_ nM	±	Ki nM	±
FhCL1	Z-Phe-Ala-CHN_2_	31.4	0.61	1.62	0.03
FhCL2	Z-Phe-Ala-CHN_2_	55.2	4.3	5.32	0.42
FhCL3	Z-Phe-Ala-CHN_2_	969.3	67.8	336.5	23.2
FhCL1	Cathepsin K inhibitor II	12.2	0.75	0.63	0.03
FhCL2	Cathepsin K inhibitor II	4.8	1.0	0.46	0.09
FhCL3	Cathepsin K inhibitor II	26.1	0.3	9.05	0.15

The inhibitor known as cathepsin K Inhibitor II (Z-LNHNHCONHNHLF-Boc, CKII) is a potent time-dependent inhibitor of human cathepsin K; its selectivity for this enzyme is largely because of the effectiveness by which Leu occupies the S2 subsite [53]. FhCL1 and FhCL2 were both potently inhibited by cathepsin K inhibitor II with Ki values of 0.63 nM and 0.46 nM respectively. In contrast, CKII was 14-fold less effective against FhCL3 (Ki 336 nM) compared to FhCL1 and 20-fold less effective compared to FhCL2 (Table 3). The data are consistent with the kinetic data for hydrolysis of peptidyl fluorogenic substrates as both FhCL1 and FhCL2 had high *k*cat/*Km* values for Z-Leu-Arg-NHMec whereas that of FhCL3 against this substrate was much lower (Table 2).

### Recombinant FhCL2 and FhCL3, but not FhCL1, cleave native collagen at physiological pH

**Figure 1:**
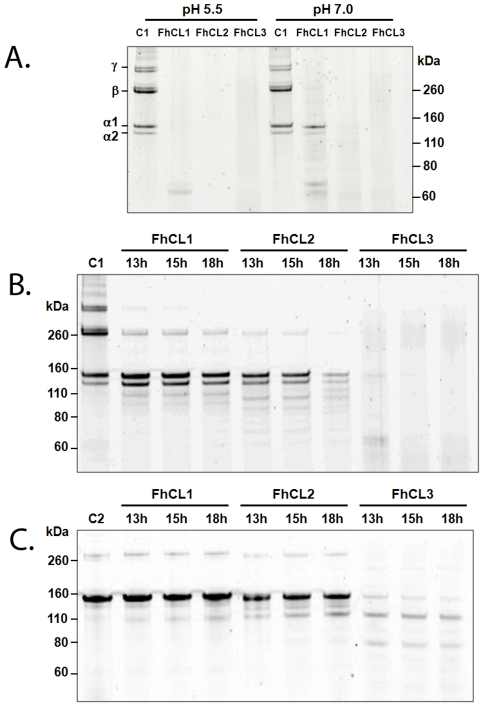
Comparison of the collagen cleaving activities of recombinant *F. hepatica* FhCL1, FhCL2 and FhCL3. (A) Type I collagen was incubated with 2 µM FhCL1, FhCL2 and FhCL3 for either 3 h (pH 5.5) or 20 h (pH 7) at 28°C. Type I collagen (B) and type II collagen (C) were incubated with 2 µM FhCL1, FhCL2 and FhCL3 at pH 7 for up to 18 h at 28°C. Reaction aliquots were analysed on 4–12% NuPage Bis-Tris gels (Invitrogen). C1, type I collagen alone; C2, type II collagen alone.

The α chains of collagens are woven together to form triple helical, or Col, regions of collagen. These are flanked by non-collagenous, or non-helical, regions termed NC domains Type I and type II collagens are most abundant in nature and are the major components of vertebrate connective tissue. They share ∼70% primary sequence identity and are composed largely of repeating Gly-X-Y motifs [27]. FhCL1, FhCL2 and FhCL3 effectively degraded type I collagen at pH 5.5 which induces a denaturation of the protein's helical Col structure. However, FhCL1 was much less able to degrade type I collagen at pH 7.0, where its native structure is preserved, and its activity was limited to the β and γ chains of the NC domains leaving the α1 and α2 chains of the Col domain intact (Fig. 1A). By contrast, both FhCL2 and FhCL3 degraded native collagen at pH 7.0 and cleaved efficiently within the Col helical structures as revealed by the breakdown of the α1 and α2 chains (Fig. 1A).

To determine the relative activity of FhCL1, FhCL2 and FhCL3 for collagen type I, digests were performed at pH 7.0 over a time course (up to 18 h) at 28°C (Fig. 1B). Only FhCL3 was capable of completely digesting collagen type I after 18 h incubation in these conditions. FhCL2 digested collagen α chains to a lesser extent than FhCL3 while FhCL1 only digested the β11 and β12 dimers but not the collagen α chains (Fig. 1B). Similarly, only FhCL3 was capable of degrading type II collagen whilst FhCL2 displayed much less activity against this substrate at pH 7.0 (Fig. 1C). FhCL1 was unable to cleave within the tightly wound type II collagen helices under these conditions (Fig. 1C).

### FhCL2 and FhCL3 cleave at different sites within the native collagen structure

**Figure 2:**
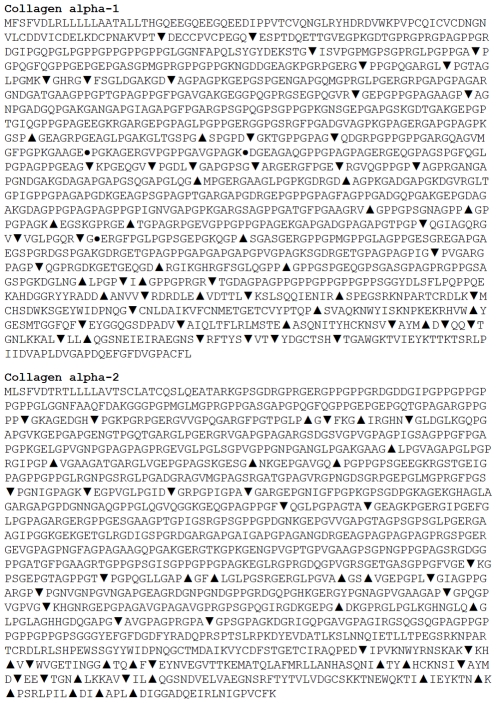
Hydrolysis of bovine type I collagen by FhCL2 and FhCL3 and analysis of digests by LC-MS/MS. Map of collagen α1 and α2 chains indicating sites of FhCL2 (triangle) and FhCL3 (inverted triangle) cleavage. Cleavage sites shared by FhCL2 and FhCL3 are shown by (circle).

To identify the cleavage sites for FhCL2 and FhCL3 within collagen type I α1 and α2 chains, the 18 h reaction aliquots (shown in Fig. 1B) were analysed by tandem mass spectrometry to determine the masses and sequence identities of the resulting hydrolytic products. Liberated peptides were mapped onto the primary amino acid sequence of bovine collagen to identify the cleavage sites of the *F. hepatica* peptidases (Fig. 2). FhCL2 cleaved collagen type I at 43 sites within the α1 chain and 26 sites within the α2 chain while FhCL3 cleaved at 24 sites within the α1 chain and 24 sites within the α2 chain. Strikingly, only three of these cleavage sites were shared between FhCL2 and FhCL3, all of which occurred in the α1 chains (Fig. 2).

**Figure 3:**
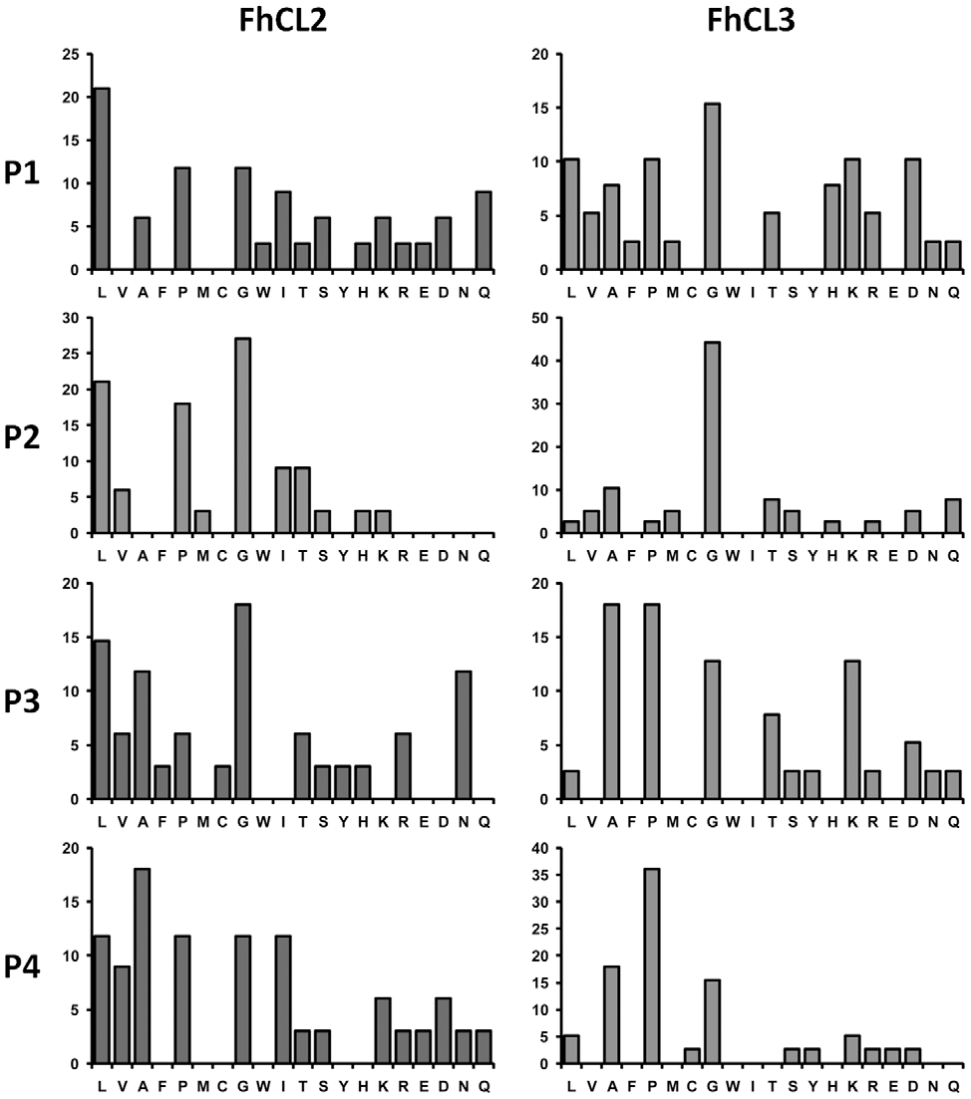
Collagen P1-P4 residues. Residues occupying the P1-P4 positions in peptides released from type I collagen following digestion by FhCL2 and FhCL3 as determined by LC-MS/MS analysis of digest samples (see Fig. 2). The frequency by which amino acids occur at the P1-P4 positions of collagen α1 and α2 chains (converted to a percentage of the total) are plotted for the 18 h reactions shown in Fig. 1B.

We examined the frequency of each amino acid in the P1, P2 and P3 position of the proteolytic cleavage sites identified in the collagen digests described above to determine preferences for binding their respective active site S1, S2 and S3 subsites (Fig. 3). While substrate residues present at the P2 position from the scissile bond interact with the S2 subsite of the active site of papain-like cysteine peptidases are considered most critical in determining the efficiency by which the P1-P1′ bond is cleaved [54], the binding of these residues are influenced by residues in the P3 positions. Consistent with our previous findings using positional scanning of synthetic combinatorial libraries the P1 position can be occupied by many different amino acids without a strong preference [22]. However, specificity is observed in the P2 position; Gly was most commonly found in the P2 position of the FhCL2 cleavages (27%), and this was followed by Leu (21%) and Pro (18%) (Fig. 3). By contrast, FhCL3 displayed a highly specific preference for Gly at the P2 position (44% of all cleavages) with a weak preference for all other amino acids including Leu and Pro in this position (3% for both residues, Fig. 3). The P3 and P4 positions were occupied by a wide range of amino acids.

**Figure 4:**
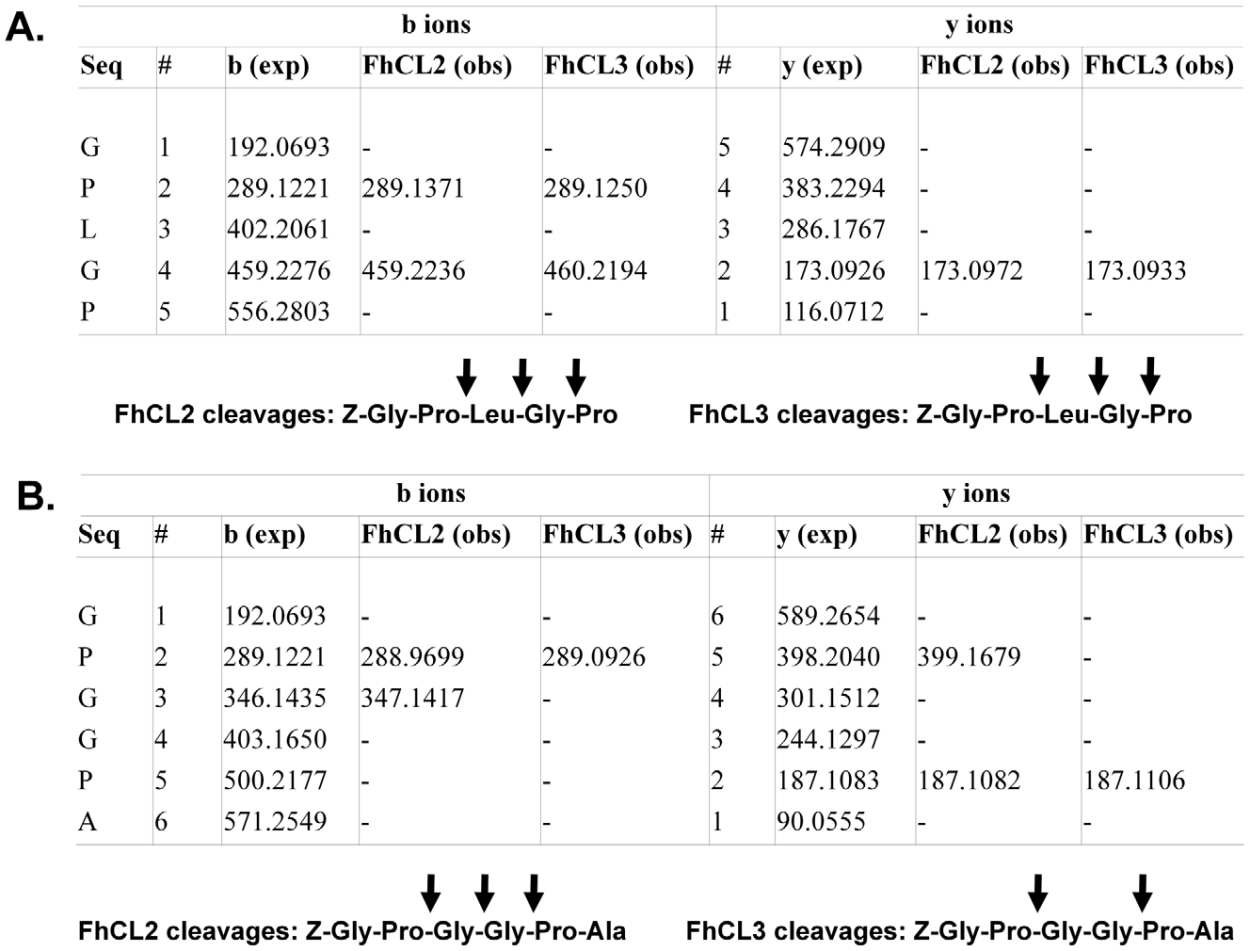
LC-MS/MS data for the cleavage of small collagen-like peptide substrates by recombinant FhCL2 and FhCL3. Substrates Z-Gly-Pro-Leu-Gly-Pro (A) and Z-Gly-Pro-Gly-Gly-Pro-Ala (B) were incubated with FhCL2 and FhCL3 for 15 min at pH 4.5 and the reactions were analysed by LC-MS/MS. The resulting peptide fragment masses (obs) that matched with y and b ions derived from theoretical fragmentation of the peptide substrates (exp) were used to map the FhCL2 and FhCL3 cleavage sites (arrows).

To further investigate the cleavage of collagen by FhCL2 and FhCL3, the ability of both enzymes to cleave two small peptide substrates, Z-Gly-Pro-Leu-Gly-Pro and Z-Gly-Pro-Gly-Gly-Pro-Ala, mimicking the repeating Gly-X-Y motifs (where X is often Pro) that occur within the collagen primary sequence was followed by tandem mass spectrometry. The presence of several peptides matching hydrolytic cleavage products showed that FhCL2 and FhCL3 were able to digest both substrates (Fig. 4). The cleavage pattern of peptide Z-Gly-Pro-Leu-Gly-Pro was identical for both FhCL2 and FhCL3. However, while FhCL2 cleaved the peptide Z-Gly-Pro-Gly-Gly-Pro-Ala at three sites (with Gly or Pro in the P2 position), FhCL3 was unable to cleave at one of these three sites where Pro occupied the P2 position (Fig. 4).

### Molecular dynamics (MD) simulations provide a structural explanation for collagen digestion by FhCL2 and FhCL3

**Figure 5:**
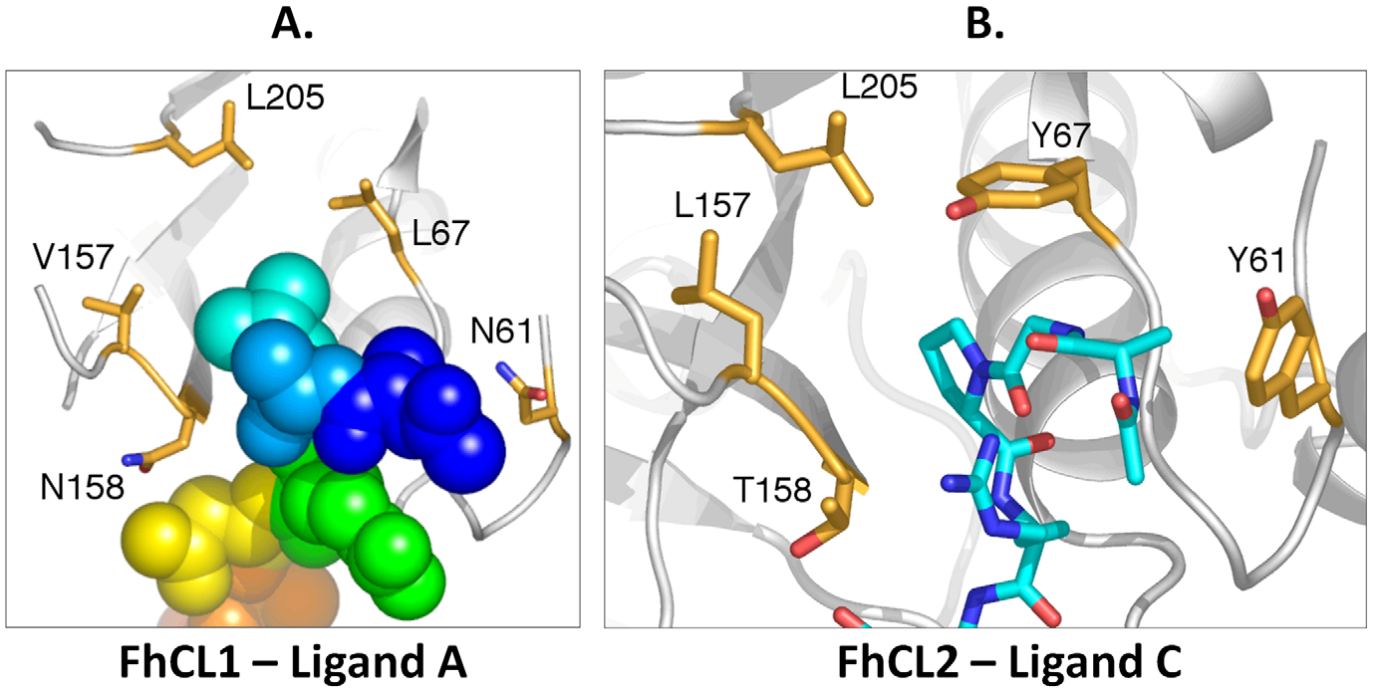
Disposition of residues within ligand binding subsites S1′, S2 and S3. (A) Final trajectory frame from the MD simulation of Ligand A (AALR*NAA) bound to wild type FhCL1, with Leu at P2. The ligand is shown in space filling representation and coloured in a spectrum pattern, with N-terminal residue 1 blue and C-terminal residue 7 red. The side-chain of P2 residue Leu (cyan) is bound in the S2 subsite. The side-chains of residues mutated in the MD simulations are shown in stick form with carbon atoms tan, oxygen red and nitrogen blue, with papain numbering. Secondary structural elements of the peptidase are shown in cartoon representation in grey. (B) Final trajectory frame from the MD simulation of Ligand C (AGPR*NAA) bound to FhCL2, with Pro at P2. Interaction of Tyr 67 with Pro bound in subsite S2 is observed. The ligand is shown in stick form with carbon atoms cyan, oxygen red and nitrogen blue.

In order to delineate the molecular basis of the ability of FhCL2 and FhCL3 to digest collagen, our recently determined crystal structure of FhCL1 was used as the starting point for a computational analysis of ligand binding. Complexes of FhCL1, with variations to key residues involved in substrate binding (summarised in Table 4), bound to different seven-residue peptides (Table 5), were analysed by performing MD simulations. Using the simulation trajectories, free energies of binding of the peptide substrates were calculated using the well-established MM-PBSA method [44], [45]. In addition, the distances between the nucleophilic sulphur atom of the active site Cys residue and the backbone carbonyl carbon atom of the scissile peptide bond, were examined over the course of the simulations. Since higher frequencies of close approach of these atoms would likely correlate with higher frequencies of formation of the transition state of the hydrolysis reaction this measure gives an indication of how well the substrate fits into the binding cleft and how readily it is cleaved [55]. Fig. 5 illustrates the critical residues of the active site investigated and their disposition in FhCL1 relative to the bound peptide substrate ligand A (AALR*NAA, shown as an example, asterisk represents position of scissile bond) and in FhCL2 bound to ligand C (AGPR*NAA). Table 5 presents the results of the binding energy calculations, as well as the average nucleophilic sulphur-scissile carbon (S-C) distances for the various peptidase-ligand complexes simulated. Fig. S1 illustrates the regions of the peptidase that contact the ligand during the FhCL1 ligand A simulation.

**Table 4:** *F. hepatica* cathepsin L variants used for the MD simulations.

Subsite	S3	S2	S2	S1′	S2	
Residue	61	67	157	158	205	
**Structure**						**Description**
FhCL1	Asn	Leu	Val	Asn	Leu	Wild-type FhCL1
FhCL2	Tyr	Tyr	Leu	Thr	Leu	FhCL2 S1′, S2, and S3 subsites
FhCL3	His	Trp	Val	Thr	Val	FhCL3 S1′, S2, and S3 subsites
HK	Asp	Tyr	Leu	Asn	Leu	Human cathepsin K
GZ	His	Trp	Ala	Asn	Phe	Ginger rhizome peptidase

FhCL1 variants used for the MD simulations and their relationship to the substrate binding subsites of FhCL2 and FhCL3 variants. Substituted residues are in red and papain numbering is used for residue positions. For reference, the equivalent residues in the collagenolytic cathepsins human cathepsin K and ginger rhizome peptidase are also given.

**Table 5:** Computed free energies of binding and average nucleophilic sulphur-Scissile carbon distances^a^.

Ligand:	AAALR*NAA	BAAPR*NAA	CAGPR*NAA	DPAGP*AGP	EPLGP*AGP	FPPGP*PGP
FhCL1 ΔGΔd	−10.83 (1.18)3.96 (0.24)	−9.10 (1.30)4.00 (0.25)		−8.30 (0.96)4.41 (0.30)		
FhCL2 ΔGΔd	−11.77 (0.75)3.97 (0.24)	−6.30 (1.19)3.95 (0.24)	−10.88 (0.96)3.94 (0.23)	−10.83 (1.30)4.27 (0.34)	−4.11 (1.02)4.82 (0.51)	−b> 10
FhCL3 ΔGΔd	−10.40 (0.84)3.99 (0.26)	−8.28 (0.82)4.06 (0.25)	−12.71 (0.95)4.05 (0.25)	−> 10	−9.56 (1.20)4.40 (0.28)	−b4.08 (0.5)

a Amino acid sequences of peptide ligands A–F are shown in single letter code with P2 in red and the position of the scissile bond shown by an asterisk; Hyp is indicated by P in ligand F. FhCL1 variants are shown in the leftmost column and are denoted as in Table 4. Mean energies (ΔG) are in kcal/mol, with corresponding standard error of the mean in parentheses. The average distances between the sulphur atom of the catalytic Cys and the backbone carbonyl carbon of scissile ligand residue 4 (Δd) are in Ångstrom, with corresponding standard deviations in parentheses. All measures calculated over the final 4.005 ns of the simulation. Standard errors of the mean for all Δd measures are <0.01 Å;

b Not calculated, see Methods.

The MD simulations indicate that, for wildtype FhCL1, activity is greatest for substrates with Leu at P2 and that Arg is favoured at P1 (consistent with our substrate and inhibitor binding kinetics shown in Table 2 and 3). Thus, the results for the FhCL1-ligand A complex (Table 4; Fig. 5A) are taken as a benchmark against which the other results are compared. The free energy of ligand binding is related to the dissociation constant K_d_ by the formula Δ*G = −RT ln K_d_*. Thus, the calculated binding energy for the FhCL1-ligand A complex of 10.83 kcal/mol corresponds to a K_d_ of 22.9 nM, while the approximate level of error in the free energy calculations of 1 kcal/mol corresponds to a 5-fold difference in K_d_. Where differences between calculated binding energies are greater than the error bounds, the calculations are taken to predict differences in binding affinities. The calculations for ligand A (AALR*NAA), which has Leu at P2, thus discriminate between binding affinities for FhCL2 and FhCL3, predicting an approximately 5–10 fold difference in K_d_, which correlates well with the inhibition constants determined for the CKII inhibitor (Table 3), which also has Leu at P2.

**Figure 6:**
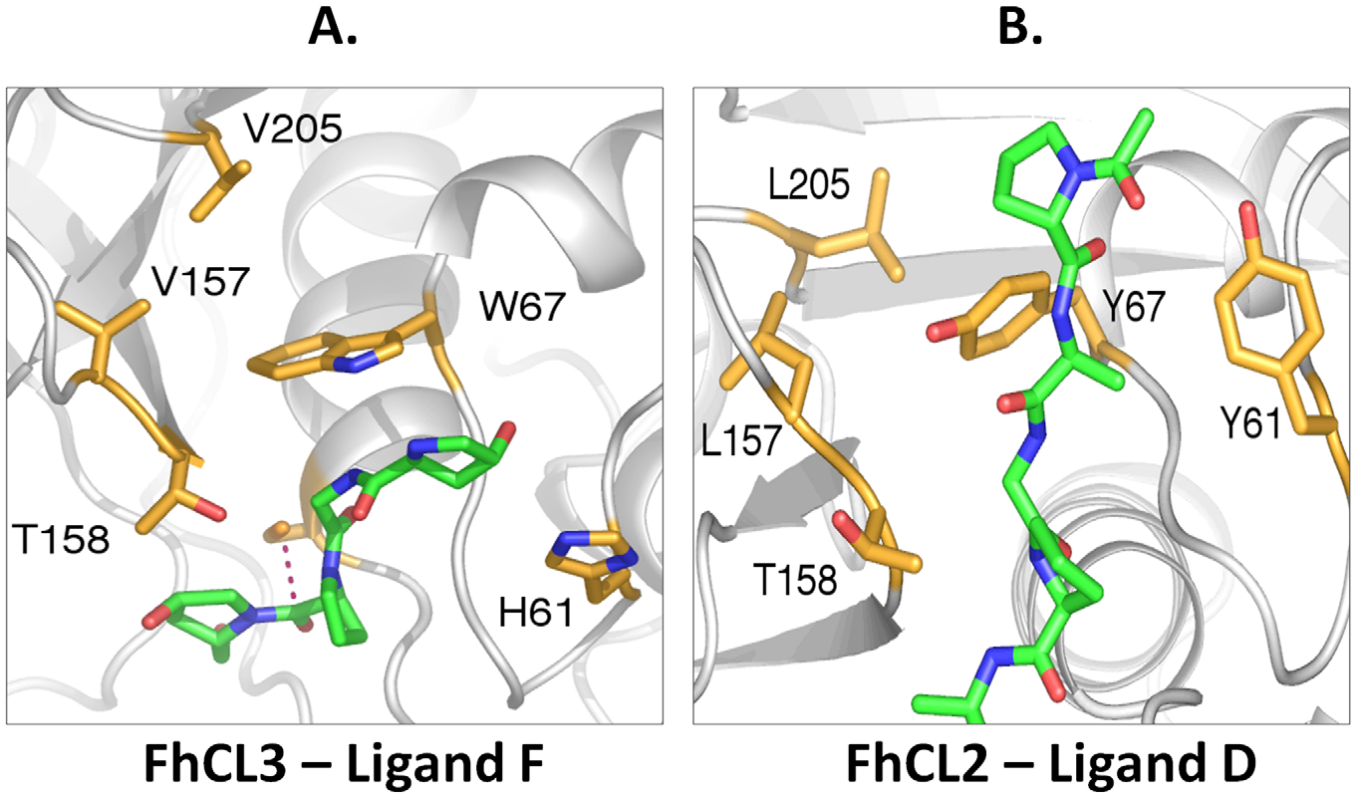
Binding mode of Gly at P2 in the FhCL2 and FhCL3 S2 subsites. The side-chains of residues mutated in the MD simulations are shown in stick form with carbon atoms tan, oxygen red and nitrogen blue, with papain numbering. The ligand is shown in stick form with carbon atoms green, oxygen red and nitrogen blue. Secondary structural elements of the peptidase are in shown in cartoon representation in grey. (A) Frame from the final 4 ns of the FhCL3-ligand F (PPGP*PGP) simulation showing the interaction of the Trp (W) 67 side-chain within the S2 subsite cleft with the ligand P2 Gly (G). The N-terminal acyl group and Pro residue of ligand F are omitted for clarity. The S-C distance is indicated by a red dashed line. (B) Final frame from the FhCL2-ligand D (PAGP*AGP) simulation showing the interaction of the Tyr (Y) 67 side-chain within the S2 subsite cleft with the ligand P2 Gly (G).

The calculations also agree with the experimental data in suggesting reduced activity of FhCL1 against a ligand with Pro at P2 (ligand B, AAPR*NAA) compared to Leu (ligand A). However, they also predict that for ligand B, FhCL1 has a higher binding affinity than FhCL2 and equal or greater activity than FhCL3. When ligand B is altered such that Gly is substituted for Ala at P3 (ligand C, AGPR*NAA), the binding affinities for the peptidases with FhCL2 or FhCL3 S2 subsites show a marked increase over those for ligand B. Thus, the data suggest that the collagenolytic activity of FhCL2 and FhCL3 may not be due simply to the P2 Pro-S2 subsite interaction, and that Gly at ligand residue P3 is also important, consistent with our earlier suggestions [23]. This inference is consistent with our previously reported experiments using combinatorial libraries [22] that showed FhCL2, strongly favoured a Gly at P3, and with our present data using native collagen digestion which indicated that Gly is favoured at P3 for both FhCL2 and FhCL3. Analysis of our collagen digest revealed that of the 11 cleavage sites for FhCL2 and FhCL3 (Tyr and Trp at position 67, respectively) containing Pro at P2 (seven for FhCL2 and four for FhCL3), 8 had Gly at P3. Given their similar active site residues to FhCL2 and FhCL3 we also analysed previous studies with human cathepsin K [56] and ginger rhizome GP2 [29] (also possess Tyr and Trp at position 67, respectively) and observed that of the 12 peptidase cleavage sites within native collagen type I containing Pro at P2 (eight for cathepsin K and four for GP2), 10 had Gly at P3. Examination of the simulation trajectories suggests that Gly (that lacks a side-chain) at P3, would offer minimal steric interference with the large active site Tyr or Trp side-chain at position 67 in FhCL2 and FhCL3, respectively, allowing the Tyr or Trp ring to form a “lid” over the ligand's P2 Pro ring, helping to sequester it in the S2 subsite (Fig. 6).

Analysis of the cleavage sites within native type I collagen show that both FhCL2 and FhCL3 have a strong preference for Gly at P2, most particularly for the latter enzyme (Figs. 2 and 3). To investigate the molecular basis of this preference, simulations were performed using ligands with Gly at P2 (ligands D and E). The simulations with Gly at P2 generally showed markedly greater S-C distances than were observed in the complexes with Leu or Pro at P2 (Table 5). This supports the idea that the P2-S2 interaction has a strong influence on the S-C interaction. The binding affinity of collagen-like ligand D (which has Ala at P3, PAGP*AGP) is substantially higher for FhCL2 compared wildtype FhCL1, but when in complex with FhCL3, ligand D essentially disengages. However, when ligand D is altered such that Leu occurs at P3 (ligand E, PLGP*AGP), binding affinity to FhCL3 is restored but greatly reduced in the complex with FhCL2. These results further support the idea that the interaction of ligand residue P3 with the peptidase is a significant factor in ligand binding, and possibly of greater importance when Gly is at P2.

**Figure 7:**
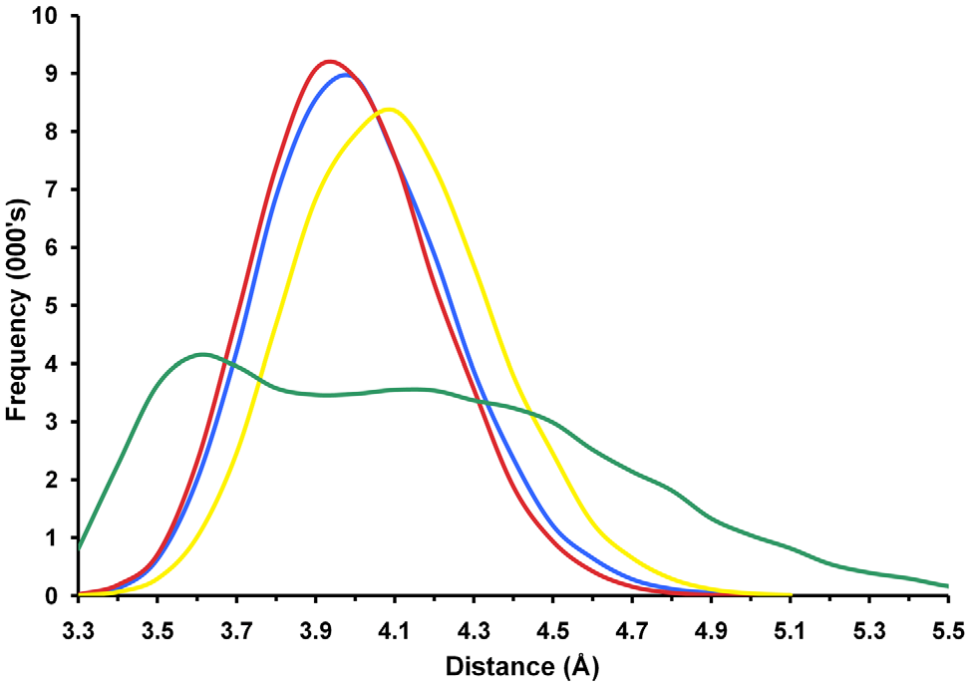
Frequency distributions of nucleophilic sulphur-scissile carbon distances. Smoothed histogram plots of the frequencies of distances between the sulphur atom of the active site nucleophilic sulphur atom and the ligand P1 backbone carbonyl carbon atom. Measures taken over the final 4 ns of each simulation at 0.075 ps intervals (53334 measures per simulation); frequencies assigned to 0.1 Å bins between 3.3 Å and 5.5 Å. Simulation FhCL1-ligand A blue, FhCL2-ligand C red, FhCL3-ligand C yellow, FhCL3-ligand F green.

Collagens comprise polypeptide chains containing the repeating triplet sequence Gly-Pro-Y where 4-hydroxyproline (Hyp) commonly occupies the Y position [57]. Thus, simulations of complexes with a ligand containing Hyp at P3 and P1′ and Gly at P2 (ligand F, PPGP*PGP) were performed. For the FhCL2 variant, the ligand began to disengage from the peptidase whilst for FhCL3 the ligand remained closely bound. Although the binding affinity for the FhCL3-ligand F complex was not calculated, the average S-C distance was much lower than for the other complexes with Gly at P2 (Table 5). Moreover, the plot of the S-C distance frequencies showed high frequencies of very close approach for the FhCL3-ligand F complex (Fig. 7). These data suggest that FhCL3 is able to digest collagen with Hyp-Gly at P3-P2 whereas FhCL2 cannot. This may explain why we observed a greater ability of FhCL3 to digest type I and II collagen compared to FhCL2 (Fig. 1).

The FhCL3-ligand F simulation trajectories revealed that the side-chain of Trp 67 occupies the FhCL3 S2 subsite and sits against the peptide backbone of the ligand P2 Gly (Fig. 6A). This may stop solvent from entering the S2 subsite and interacting with the ligand P2 Gly, thus “sealing” the ligand in the enzyme's binding cleft. A similar disposition of the Trp 67 side-chain was also observed in the FhCL3-ligand E simulations. Tyr at position 67 in FhCL2 behaves in a similar manner to the Trp 67 of FhCL3 when binding ligands with Gly at P2. Thus, Tyr 67 occupied the S2 subsite cleft and contacted the peptide backbone of the ligand P2 Gly in the FhCL2-ligand D complex (Fig. 6B). The position of the Tyr side-chain was further stabilised by a hydrogen bond between its hydroxyl oxygen and the backbone oxygen of residue 157.

*F. hepatica* has evolved a repertoire of cathepsin L peptidases as a result of gene duplication and diversification that exhibit subtle but distinct substrate specificities [1], [8], [14], [15]. The expression of different members of this peptidase family is temporally regulated suggesting that they perform precise functions at different stages of the parasites' development [13]. This idea is supported by our previous data showing that the predominant enzyme, FhCL1, secreted by the mature adult parasites, which are obligate blood-feeders, is adapted to the degradation of host haemoglobin; the S2 subsite of the FhCL1 active site, which contributes mostly to substrate binding, readily accommodates P2 residues such as Leu, Ala, Val and Phe that together represent >40% of the residues present in haemoglobin [3].

FhCL1 does not readily accept Pro into the S2 subsite as shown in this and other [22], [28] studies and thus it's activity against type I and II collagens observed here was restricted to the non-collagenous, NC, domains. By contrast, both FhCL2 and FhCL3 have evolved to accommodate Pro in the S2 subsite of their active sites; this property has been attributed to the presence of Tyr and Trp, respectively, at position 67 within the S2 subsite of these enzymes, a position that is occupied by Leu in FhCL1 [1], [14], [15], [22], [23]. In this study, our computational data show that Tyr and Trp at position 67 have the ability to function in distinct ways to accommodate either Gly or Pro residues at P2 and explains why FhCL2 and FhCL3 have an ability to degrade the Gly-X-Y containing Col helices of collagen. The results are also in accordance with our previous suggestion that the interaction of substrate residue P3 with the peptidase is a significant factor in substrate binding, in particular when Gly or Pro is at P3 [23]. This is also the case when we compare FhCL3 specificity towards synthetic peptides; Pro is readily accepted in P2 only when Gly is at P3 (Tos-Gly-Pro-Arg-NHMec, Tos-Gly-Pro-Lys-NHMec and Boc-Ala-Gly-Pro-Arg-NHMec) but not when Val is at P3 (Boc-Val-Pro-Arg-NHMec) (Table 2). Therefore, the collagenolytic activity of FhCL2 and FhCL3 is not due simply to the P2 Pro-S2 subsite interaction, and with Pro at P2, Gly at residue P3 is critical. A comparison of the cleavage sites of FhCL2, FhCL3, human cathepsin K [56] and ginger rhizome GP2 [29] revealed that many of their cleavage sites within collagen where Pro is at P2, a Gly is present at P3. A P3 Gly, which lacks a side-chain, offers minimal steric interference with the large active site Tyr or Trp side-chain at position 67, and allows the Tyr or Trp ring to form a “lid” over the ligand's P2 Pro ring, helping to sequester it in the S2 subsite (Fig. 6).

However, we observed that FhCL3 digested type I and II collagens more efficiently compared to FhCL2 and that these two enzymes cleave at mostly different sites (see Figs. 1 and 2). The computational data indicates that this may be, in part, because FhCL3 binds substrates containing a P3 and P1′ Hyp much tighter than FhCL2. A difference between these two enzymes was also observed using a peptide substrate that mimics the Gly-X-Y repeat in the collagen Col domain, Z-Gly-Pro-Gly-Gly-Pro-Ala; FhCL2 cleaves at three sites with Gly or Pro in the P2 position, whereas FhCL3 was unable to cleave at one of these three sites despite having Pro occupying the P2 position and Gly at the P1 position. This result contrasts with our data using fluorogenic peptide substrates which showed that FhCL3 cleaved the tripeptides Tos-Gly-Pro-Arg-NHMec and Tos-Gly-Pro-Lys-NHMec with 5- and 3-fold better efficiency, respectively, than FhCL2. On the other hand, the two enzymes exhibited equal efficiency for the substrate Boc-Ala-Gly-Pro-Arg-NHMec. The influence of P4 and P′ regions of the peptides on substrate binding in these two enzymes need greater attention in future studies when suitable reagents become available. Notwithstanding, it is clear that the modification within the active site of FhCL2 and FhCL3 (Tyr or Trp at position 67) has subtly altered the substrate specificity of the two enzymes such that they exhibit different substrate profiles without compromising their unique ability to degrade host native collagen.

FhCL3 is expressed by the invasive stage of *F. hepatica* which must quickly penetrate the wall of the intestine to enter its host [1], [13]. RNAi-mediated knockdown experiments have demonstrated that the secretion of this peptidase and a cathepsin B cysteine peptidase by these invasive parasites is critical to invasion of the intestinal tissue [2]. Once the intestine has been traversed expression of these enzymes is switched off and the parasite up-regulates expression and secretion of FhCL1 and FhCL2 which are required to facilitate tunnelling through the liver mass and feeding on host tissue (the parasite undergoes rapid growth at this stage) [16]. The collagenolytic activity of FhCL3 and FhCL2 is important in degrading the extracellular matrix of the tissues through which this parasite moves. While collagenase activity has been demonstrated in ginger rhizome cysteine peptidases [29], [58], only one other animal cysteine peptidase, human cathepsin K which functions in bone re-modelling [59], possesses collagenase activity. Accordingly, the evolution of this activity in *F. hepatica* must represent an important step in the development of a parasitic way of life.

## Supporting Information

Figure S1 **Residues contacting the ligand.** Final trajectory frame from the MD simulation of Ligand A (AALR*NAA) bound to wild type FhCL1, with Leu at P2. The ligand is shown in stick form with carbon atoms magenta, oxygen red and nitrogen blue. The side-chain of P2 residue Leu (cyan) is bound in the S2 subsite. Secondary structural elements of the protease in cartoon representation and the side-chains of residues that contact the ligand in the MD simulations shown in stick form. Sidechain atoms or backbone segments of the peptidase which contacted the ligand are coloured according to the relative frequency of contact over the final 4 ns with yellow (low) to green (high).
(TIF)
