# Supplementary figures and images for: Collagenolytic Activities of the Major Secreted Cathepsin L Peptidases Involved in the Virulence of the Helminth Pathogen, Fasciola hepatica
Source: PLoS Negl Trop Dis. 2011 Apr 5;5(4):e1012. doi: 10.1371/journal.pntd.0001012 (PMC3071364; doi:10.1371/journal.pntd.0001012)

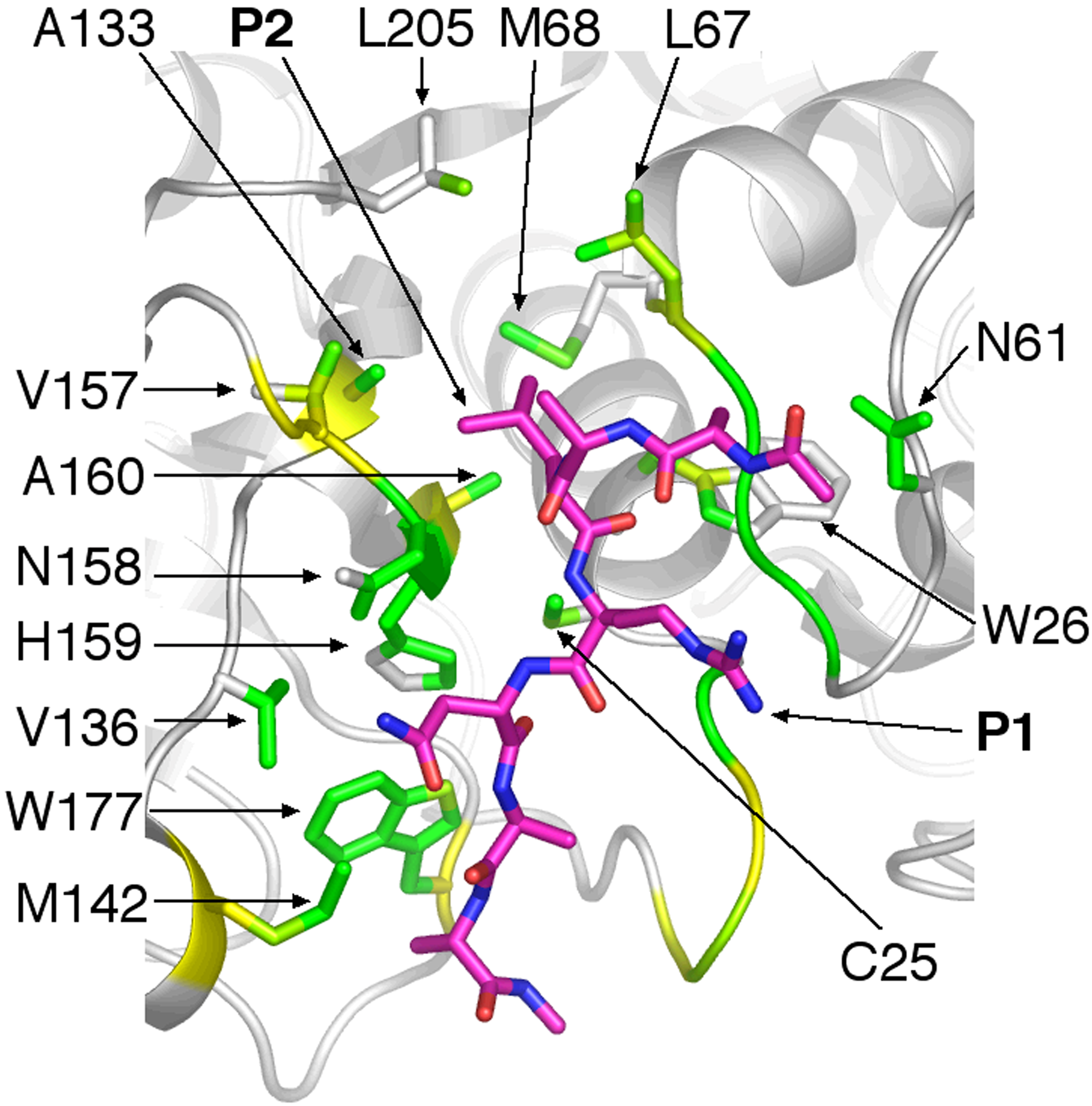

Supplement: Figure S1 — Residues contacting the ligand. Final trajectory frame from the MD simulation of Ligand A (AALR*NAA) bound to wild type FhCL1, with Leu at P2. The ligand is shown in stick form with carbon atoms magenta, oxygen red and nitrogen blue. The side-chain of P2 residue Leu (cyan) is bound in the S2 subsite. Secondary structural elements of the protease in cartoon representation and the side-chains of residues that contact the ligand in the MD simulations shown in stick form. Sidechain atoms or backbone segments of the peptidase which contacted the ligand are coloured according to the relative frequency of contact over the final 4 ns with yellow (low) to green (high). (TIF) [file pntd.0001012.s001.tif]
